# The De Garengeot hernia: A case report of an unusual presentation of appendicitis

**DOI:** 10.1016/j.ijscr.2020.08.053

**Published:** 2020-09-24

**Authors:** Davek Sharma, Jacob Katsnelson, Emmanuel Nwachuku, Jeffrey Kolff

**Affiliations:** Department of General Surgery, Abington Hospital, Jefferson Health, 1200 Old York Rd., Abington, PA, 19001, United States

**Keywords:** De Garengeot, Hernia, Femoral, Appendix, Appendicitis, Incarcerated

## Abstract

**Introduction:**

A De Garengeot hernia is defined by a femoral hernia containing the appendix. Acute appendicitis within a femoral hernia is an extremely rare surgical presentation and occurs in only 0.08–0.013% of cases as cited by the literature.

**Presentation of case:**

A 64-year-old female presented to the Emergency Department of our hospital with acute onset of a right-sided groin bulge that occurred earlier that day after doing heavy lifting. Her workup revealed acute appendicitis contained within an incarcerated right femoral hernia. The patient underwent laparoscopic appendectomy with open femoral hernia repair. Intraoperatively, the appendiceal tip was incarcerated within the hernia sac. It was removed through the open inguinal incision after the appendix base was divided laparoscopically. Final pathology showed inflamed acute appendicitis without evidence for neoplasm.

**Discussion:**

Physicians should be aware of the rare entity of an unusual presentation of appendicitis as well as surgical options for treatment. The literature does not conclude upon a gold standard for method of approach.

**Conclusion:**

De Garengeot hernia remains a rare and unusual surgical presentation of femoral hernia, and complication of the case by incarceration leading to acute appendicitis provides a challenging surgical approach which should be individualized to each patient.

## Highlights

• DeGarengeot hernia is still defined as a rare entity in the literature.
• There is no uniform consensus on surgical management of this rare entity.
• Surgical management can be tailored on a case-by-case basis.

## 1 Introduction

Femoral hernias occur as a weakness and protrusion of abdominal viscera or contents through the femoral canal, bordered superiorly by the inguinal ligament and laterally by the femoral vessels. The femoral hernia is much more common in women and accounts for 3% of all hernias [1]. Due to its narrow neck, it has a much larger chance of strangulation compared to inguinal hernias and all are recommended for repair [1,2]. The migration of the appendix into the inguinal hernia sac is known as an Amyand hernia and is very rare. However, even more rare is the migration of the appendix into the femoral hernia sac, which is called De Garengeot hernia, with a reported incidence of 0.5–5% of all femoral hernias [2]. The incidence of De Garengeot’s hernia cited in the literature is fewer than 100 cases [2]. The incidence of concurrent appendicitis in the presence of a De Garengeot hernia is cited to be as low as 0.08%–0.13% [3]. We present a rare case of an acutely inflamed appendicitis within a De Garengeot hernia diagnosed preoperatively and managed surgically.

## 2 Case

This case report has been written in accordance with the SCARE 2018 criteria [4].

A 64-year-old female presented to the Emergency Department of our hospital with acute onset of a right-sided groin bulge that occurred earlier that day after doing heavy lifting. She had no significant past medical or surgical history. Her baseline functional status was exceedingly well. Her laboratory studies showed no leukocytosis or any electrolyte abnormalities. Initial radiologic studies included a transabdominal and transvaginal pelvic ultrasound, which showed a 2.4 × 2.6 cm fluid collection with some septations and absent visualized adnexal structures. A subsequent CT demonstrated a well-circumscribed 2.7 × 3.2 × 2.6 cm fluid collection containing echogenic debris. It did not have significant rim enhancement yet was noted to be most consistent with an abscess, complex seroma, or necrotic lymph node; its depth was amenable to percutaneous drainage. On further review with the radiologist, it was noted to contain the appendix, the tip of which was dilated and radiographically concerning for a cystic process, with mucocele remaining within the differential diagnosis.

**Fig. 1:**
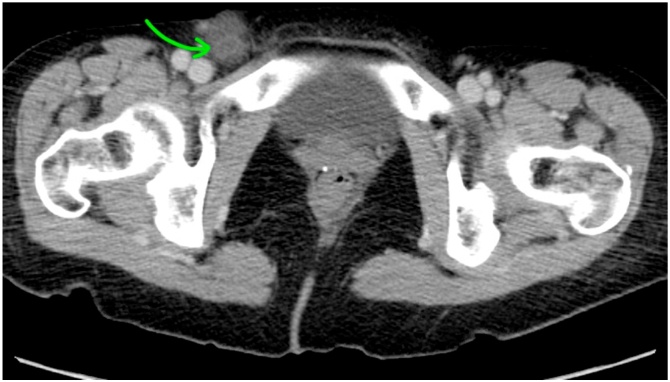
Computed tomography scan demonstrating right-sided inguinal hernia—in this case, a dilated appendiceal tip within the femoral canal (De Garengeot hernia).

**Fig. 2:**
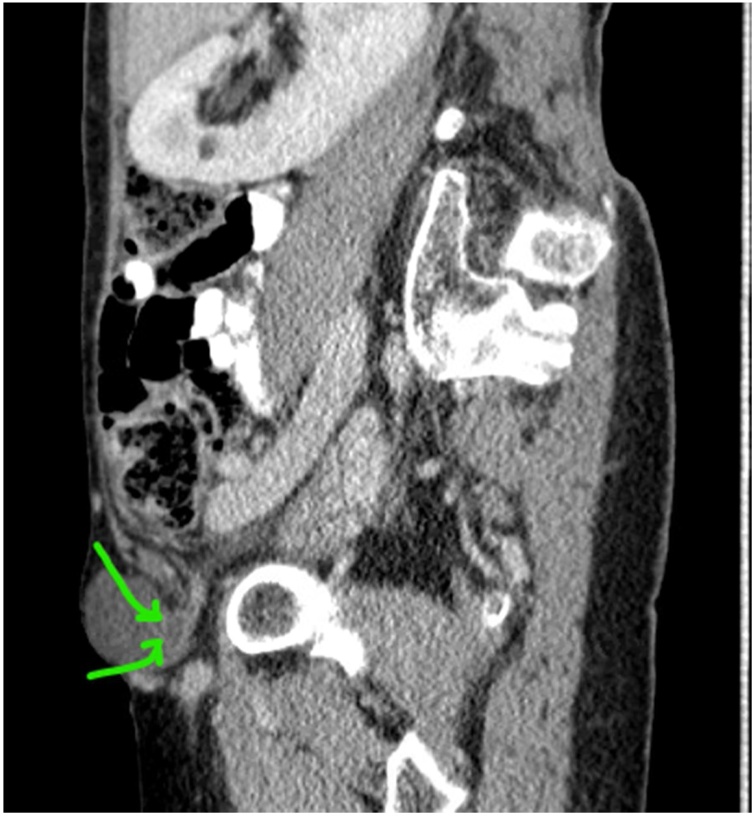
Sagittal view showing appendiceal tip within De Garengeot hernia.

The patient was kept nil per os, intravenous fluid resuscitation was initiated, and antibiotic therapy was begun. She was brought to the operating room for emergent surgical intervention. The patient underwent laparoscopic appendectomy with open femoral hernia repair. Intraoperatively, the appendiceal tip was incarcerated within the hernia sac (Fig. 1). Due to the preoperative radiographic appearance and the suspicion for mucocele, extreme caution was exercised to avoid rupture and not to provide excess tension in an attempt to reduce it laparoscopically. It was easily removed through the open inguinal incision after the appendix base was divided laparoscopically (Fig. 2). The femoral hernia was repaired in standard McVay fashion. Final pathology showed inflamed non-perforated acute appendicitis without evidence for neoplasm. The patient recovered well without notable complications and was discharged on postoperative day number two without antibiotics.

## 3 Discussion

The De Garengeot hernia was first described in 1731 by French surgeon Rene Jacques de Garengeot [3]. The leading theories behind the presence of appendix within the femoral canal emphasize an embryologic abnormal pelvic appendiceal location on the cecum, predisposing it to femoral canal herniation [3,5], and a larger cecum which causes the appendix to herniate by mass effect [3]. The presence of appendicitis within the De Garengeot hernia is also debated with regards to the sequence [5]. Most seem to favor a theory of appendicitis resulting from extrinsic compression of the appendiceal lumen by a narrow femoral neck, as opposed to appendicitis happening first and then migrating down into the femoral hernia [5].

**Fig. 3:**
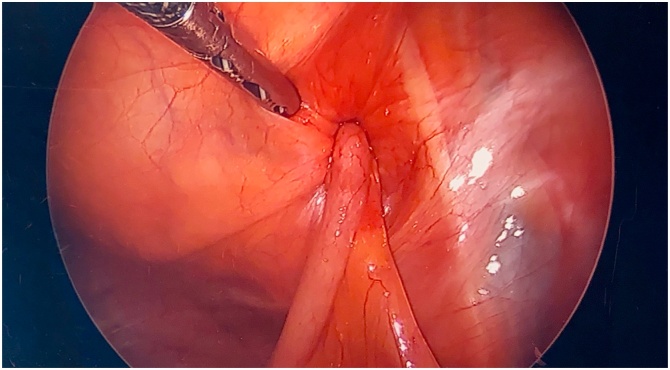
Laparoscopic peritoneal view of incarcerated appendix within the femoral canal (De Garengeot hernia).

**Fig. 4:**
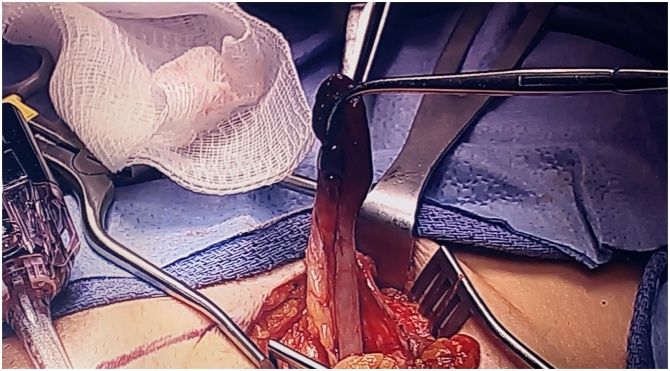
Open femoral hernia repair reveals a hernia sac containing a necrotic tip consistent with likely strangulation resulting in appendicitis.

Similar to inguinal hernia, De Garengeot hernia clinically presents with a groin lump, which is nonreducible and tender to palpation [[2], [3], [4]]. Fever or bowel obstruction are possible but rare (Fig. 3, Fig. 4).

The diagnosis is usually made preoperatively with modern imaging techniques, but there are reported cases in the literature of diagnosis being made intraoperatively [6]. Treatment is a surgical emergency. There is no specific agreed upon surgical procedure for management [6,7]. For a noninflamed appendix, laparoscopic appendectomy with intraperitoneal mesh repair might be a consideration. Beysens et al. reported the first case of laparoscopic appendectomy followed by TEP femoral hernia repair [8]. We recommend avoidance of mesh-based repair techniques in the setting of appendicitis, specifically perforated cases. Advocates of pure laparoscopic approaches support potential advantage in a shorter hospital stay and speedier recovery [9]. The most common complications are wound infections [5].

De Garengeot hernias are rare, and a concomitant diagnosis of acute appendicitis within the hernia is exceedingly rare. We believe the rarity of this case can contribute to the surgical literature to provide our institutional experience and allow practitioners to be prepared with their own thoughts for management should they ever encounter this rare surgical entity.

## 4 Conclusion

De Garengeot hernia remains a rare and unusual surgical presentation of femoral hernia, and complication of the case by incarceration leading to acute appendicitis provides a challenging surgical approach which should be individualized to each patient. As there are no guidelines for surgical management for this exceedingly rare entity, knowledge of the condition and reporting of presentations and treatments provides the surgical community with knowledge for potential approaches.

## Funding

None.

## Ethical approval

We did not seek IRB approval for this case report, which contains only retrospective, deidentified patient information. The writing or publication of this case report did not affect this patient’s treatment or outcomes in any way. There are no ethical dilemmas with this case.

## Consent

Written informed consent was obtained from the patient for publication of this case report and accompanying images. A copy of the written consent is available for review by the Editor-in-Chief of this journal on request.

## Registration of research studies

1. Name of the registry: N/A.
2. Unique identifying number or registration ID: N/A.
3. Hyperlink to your specific registration (must be publicly accessible and will be checked): N/A.

## Guarantor

Davek Sharma MD.

Jeffrey Kolff MD, FACS.

## Provenance and peer review

Not commissioned, externally peer-reviewed.

## CRediT authorship contribution statement

**Davek Sharma:** Conceptualization, Methodology, Writing - original draft. **Jacob Katsnelson:** Investigation, Methodology. **Emmanuel Nwachuku:** Writing - review & editing. **Jeffrey Kolff:** Supervision.

## Declaration of Competing Interest

The authors report no declarations of interest.

## References

[1] Fousekis F.S., Christou P.A., Gkogkos S., Aggeli P., Pappas-Gogos G. (2018). A case of De Garengeot’s hernia with acute appendicitis and literature review. Int. J. Surg. Case Rep..

[2] Talini C., Oliveira L.O., Araújo A.C., Netto F.A., Westphalen A.P. (2015). De Garengeot hernia: case report and review. Int. J. Surg. Case Rep..

[3] Bidarmaghz B., Tee C.L. (2017). A case of De Garengeot hernia and literature review. BMJ Case Rep..

[4] Agha R.A., Borrelli M.R., Farwana R., Koshy K., Fowler A., Orgill D.P., For the SCARE Group (2018). The SCARE 2018 statement: updating consensus Surgical CAse REport (SCARE) guidelines. Int. J. Surg..

[5] Sinraj A.P., Anekal N., Rathnakar S.K. (2016). De Garengeot’s hernia—a diagnostic and therapeutic challenge. J. Clin. Diagn. Res..

[6] Akbari K., Wood C., Hammad A., Middleton S. (2014). De Garengeot’s hernia: our experience of three cases and literature review. BMJ Case Rep..

[7] Freeman K.S., Picard M.M., Kovacs M.D. (2018). Acute appendicitis involving a De Garengeot hernia. J. Comput. Assist. Tomogr..

[8] Beysens M., Haeck L., Vindevoghel K. (2013). Laparoscopic appendectomy combined with TEP for de Garengeot hernia: case report. Acta Chir. Belg..

[9] Misiakos E.P., Paspala A., Prodromidou A., Machairas N., Domi V., Koliakos N., Karatzas T., Zavras N., Machairas A. (2018). De Garengeot’s hernia: report of a rare surgical emergency and review of the literature. Front. Surg..

